# Age-specific locomotor response to nicotine in yellow and mottled yellow A^vy^/a mice

**DOI:** 10.1186/1756-0500-6-497

**Published:** 2013-12-01

**Authors:** Marc A Dingman, Joseph P Gyekis, Courtney A Whetzel, Laura Cousino Klein, David J Vandenbergh

**Affiliations:** 1 Department of Biobehavioral Health, The Pennsylvania State University, 219 Biobehavioral Health Building, 16802 University Park, PA, USA; 2 Neuroscience Program, The Pennsylvania State University, 219 Biobehavioral Health Building, 16802 University Park, PA, USA; 3 Genetics Program, Chandlee Laboratory, The Pennsylvania State University, 219 Biobehavioral Health Building, 16802 University Park, PA, USA

**Keywords:** Nicotine, Agouti viable yellow, Melanocortin receptor 4, Locomotion, Thigmotaxis, Corticosterone, Hypothalamic-pituitary-adrenal

## Abstract

**Background:**

Most *Agouti viable yellow* (*A*^*vy*^) mice display constitutive expression of *agouti* protein, which acts as an inverse agonist at the melanocortin receptor 4 (Mc4r), resulting in adult-onset obesity as well as an altered sensitivity to some drugs of abuse. We investigated the influence of excessive *agouti* expression on open-field locomotor response to daily 0.5 mg/kg (-)-freebase nicotine injections in 27 early adolescent and 27 young adult male *A*^*vy*^*/a* and *a/a* mice, and assessed the effects of nicotine administration (0.5 mg/kg) followed by open-field testing on serum corticosterone levels in a separate group of 25 young adult male *A*^*vy*^*/a* and *a/a* mice.

**Findings:**

Young adult *A*^*vy*^*/a* mice displayed pronounced nicotine-induced hypolocomotion (a 24% reduction in distance traveled) compared to their *a/a* littermates. Early adolescent *A*^*vy*^*/a* mice did not differ from their *a/a* littermates or saline-matched controls in locomotion following nicotine administration. Young adult *A*^*vy*^*/a* mice also displayed increased thigmotaxis (a 5% increase in time spent outside the center of the apparatus) on the first day of nicotine administration as compared to saline-matched controls, while *a/a* mice did not. An increase in serum corticosterone levels 20 minutes after nicotine injection in a separate group of young adult male mice (n = 25) was proportionally similar between *A*^*vy*^*/a* and *a/a* mice.

**Conclusions:**

Overall, the results suggest an age- and epigenotype- or genotype-specific response to nicotine administration in young adult male *A*^*vy*^*/a* mice. It appears the *A*^*vy*^*/a* locomotor and thigmotaxic responses to acute nicotine administration are not mediated solely by hypothalamic-pituitary-adrenal (HPA) axis stimulation.

## Findings

### Introduction

In wild-type (WT) mice, the *agouti* gene is transiently expressed during hair growth and encodes a signaling molecule that creates a subapical yellow band on each, otherwise brown or black, hair [1]. This yellow and black amalgamation results in the agouti coat color of the WT mouse. *A*^*vy*^*/a* mice have a dominant mutation due to a retrotransposon insertion in the *agouti* allele that results in constitutive *agouti* expression, leading to the expression of yellow fur in the majority of animals [2]. Other littermates with the background genotype (*a/a* homozygotes) have a loss-of-function mutation in the *agouti* gene, which results in black coats [3]. In yellow and mottled yellow *A*^*vy*^*/a* mice, the *agouti* gene is expressed ectopically in all tissues, and this over-expression is associated with a phenotype that includes obesity, diabetes, and tumorigenesis [4]. *a/a* mice, on the other hand, have no ectopic expression of agouti, and thus do not display obesity or the other comorbidities that their yellow *A*^*vy*^*/a* littermates do [5]. *Agouti* expression appears to be modified epigenetically in *A*^*vy*^*/a* mice, where hypomethylation at the *Agouti* locus is correlated with a higher proportion of yellow fur, and the concomitant metabolic syndrome, while methylation at the locus results in a pseudoagouti (similar to WT) coat color and an absence of the metabolic syndrome [6].

The adult-onset metabolic syndrome in *A*^*vy*^*/a* mice is thought to be mediated at least in part by the *agouti* protein acting as an inverse agonist at the Mc4r [7], which plays an important role in feeding inhibition [8,9]. Interestingly, the melanocortin system also appears to be involved in the response to some addictive drugs. For example, melanocortins antagonize the neurobiological effects of opiates, and down regulation of Mc4rs may be integral to the development of opiate addiction [10]. Mc4rs also may be associated with the rewarding effects of cocaine, as chronic cocaine administration increases Mc4r expression in the adult male rat striatum via a dopamine-dependent mechanism [11]. Male *A*^*y*^ mice (which also exhibit melanocortin receptor antagonism) demonstrate a reduced locomotor response to repeated cocaine administration compared to C57BL/6 (B6) mice, which suggests that melanocortin receptors may contribute to the behavioral effects of cocaine [12]. Additionally, Mc4r agonists like melanotan increase ethanol intake in adult male and female rats, while Mc4r antagonists decrease intake [13].

Nicotine also may interact with or act on the same neurobiological systems as drugs like morphine [14], cocaine [15], and alcohol [16]. The result of these interactions or similarities in mechanism can sometimes be very apparent in the epidemiology of human drug use. For example, rates of cigarette smoking are often much higher in cocaine-dependent [17] individuals and alcohol-dependent [18] individuals.

Little research has been reported, however, on the relationship between nicotine and the melanocortin system besides investigations into the role melanocortins may play in the anorectic effects of nicotine e.g. [19]. To examine how melanocortin receptor antagonism might influence behavioral responses to nicotine in *A*^*vy*^*/a* mice, we repeatedly administered 0.5 mg/kg (-)-freebase nicotine to young adult and early adolescent male *A*^*vy*^*/a* mice as well as their *a/a* littermates, and measured their locomotion in an open-field apparatus. The obesity phenotype in *A*^*vy*^*/a* mice emerges in late adolescence to early adulthood, which suggests there may be age-related differences in the effect of constitutive *agouti* expression across the *A*^*vy*^*/a* lifespan [20]. Thus, we expected there might be informative changes in locomotor responses to nicotine in early adolescent versus young adult mice. After observing hypolocomotion following nicotine administration in young adult *A*^*vy*^*/a* mice, we further explored this age-specific effect by analyzing the time spent in the center vs. the periphery of the apparatus in young adult mice to determine thigmotaxis (the propensity to stay close to the walls of an open field), a purported measure of rodent anxiety [21]. Based on the results of thigmotaxis tests, nicotine administration seemed to have an anxiogenic effect on young adult *A*^*vy*^*/a* mice on the first day of treatment. Because anxiogenic events in mice are often associated with corticosterone secretion via hypothalamic-pituitary-adrenal (HPA) axis activation [22], we hypothesized that this anxiogenic effect of nicotine might be associated with increased corticosterone secretion as well. To test this hypothesis, we assayed serum corticosterone in a subgroup of young adult male *A*^*vy*^*/a* and *a/a* mice following their first exposure to nicotine.

### Methods

Seventy-nine male *A*^*vy*^*/a* (*n* =39) and *a/a* mice (*n* =40) were descendants of mice provided by Dr. Randy Jirtle (Duke University, Durham, NC). All *A*^*vy*^*/a* mice had either yellow or mottled yellow coat colors. After weaning at postnatal day (PND) 28, adolescents (*n* = 27) began behavioral testing at 29–31 PND, while behavioral testing for young adult mice (*n* = 27) took place between PND 50 and PND 70. This age range was chosen because by this point *A*^*vy*^*/a* mice have begun to display much greater weight gain than siblings that don’t have the *A*^*vy*^ mutation [15]. Thus, we hypothesized that age-related differences in the effect of constitutive *agouti* expression may emerge during this time period. During behavioral testing, animals received a subcutaneous (s.c.) injection of 0.5 mg/kg (-)-freebase nicotine in 0.9% saline or vehicle 5 minutes before being placed in the testing apparatus, a 40 × 40 × 40 cm white activity box. The dosage of 0.5 mg/kg was chosen because it is generally well tolerated by mice and results in mild changes in locomotion [23]. Animals were tested within an hour of onset of the dark cycle under dim red lights. Mice were habituated to the apparatus for 3 consecutive days by being placed in the box and allowed to explore for 15 minutes. Then, for 2 days mice received saline injections (s.c.) before being placed in the apparatus for 15 minutes to habituate to the injection procedure. For the 5 treatment days, mice received either saline or nicotine and were placed in the apparatus for 15 minutes. Activity was monitored using video tracking software (Anymaze 4.20, Stoelting, USA). Locomotor response to nicotine was measured by mean distance traveled in meters. Thigmotaxis was measured in young adult animals by assessing the distance traveled outside the center of the apparatus and dividing by the total distance traveled [24]. This calculation was used to control for the effects a reduction in locomotion itself might have on thigmotaxis. The center region of the apparatus was defined as the central 20 × 20 cm area.

Corticosterone assessment also took place between PND 50 and PND 70 in a separate group of young adult male mice (*n* = 25). These mice were sacrificed via cervical dislocation following their first nicotine injection (all mice received nicotine treatment) and subsequent 15-minute locomotion assay; trunk blood was collected. Samples sat at room temperature for 15 minutes, then were centrifuged at 1500 × g. Serum was aliquoted and frozen at minus 80°C until later assay for corticosterone. Corticosterone was determined using an enzyme immmunosorbent assay (EIA; Arbor Assays, Ann Arbor, MI, USA) in the Biomarker Core Laboratory in the Department of Biobehavioral Health at The Pennsylvania State University. The assay sensitivity was 18.6 pg/mL; the limit of detection was 16.9 pg/mL.

Locomotion was analyzed using a 4-way repeated measures ANOVA for Age x Genotype x Treatment x Treatment Day. A 3-way repeated measures ANOVA for Genotype x Treatment x Treatment Day was used to analyze thigmotaxis in young adult animals. Serum corticosterone levels were analyzed using a 2-way ANOVA for Genotype x Treatment. Differences between saline and drug-treated controls were tested using 2-sample t-tests. Findings were considered statistically significant at *p* < 0.05. Data were analyzed using SAS (version 9.2; Cary, North Carolina) statistical software.

All animal experiments were approved by the Pennsylvania State University Institutional Animal Care and Use Committee (IACUC #’s 29898 & 33133).

### Results

**Figure 1:**
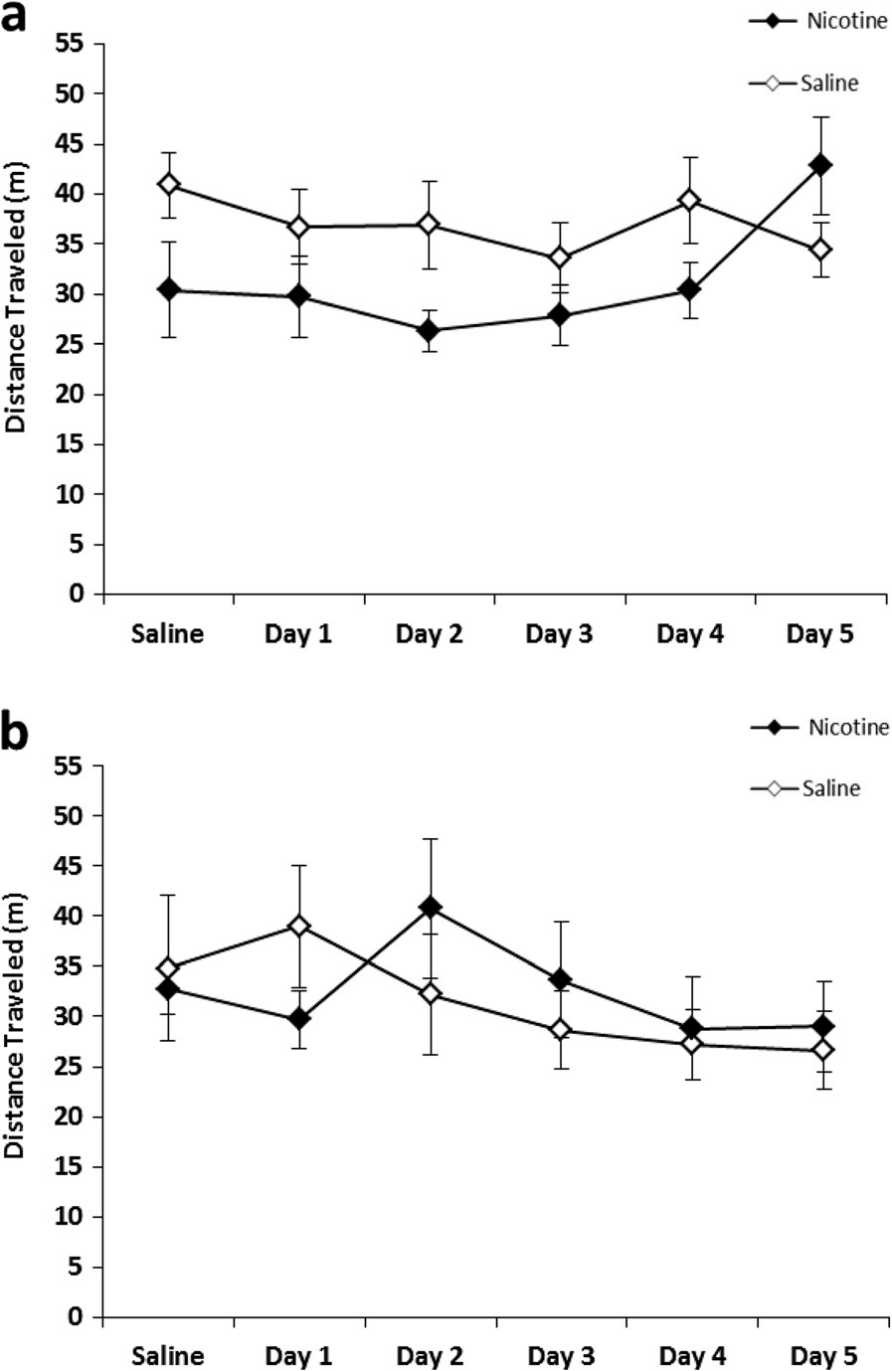
**Adolescent locomotion following nicotine administration.** Nicotine (0.5 mg/kg) was administered for 5 consecutive days to *a/a* mice **(a)**, and *A*^*vy*^*/a* mice **(b)**. Data are presented as mean distance traveled in meters ± SE; *n* = 6–8 per group.

**Figure 2:**
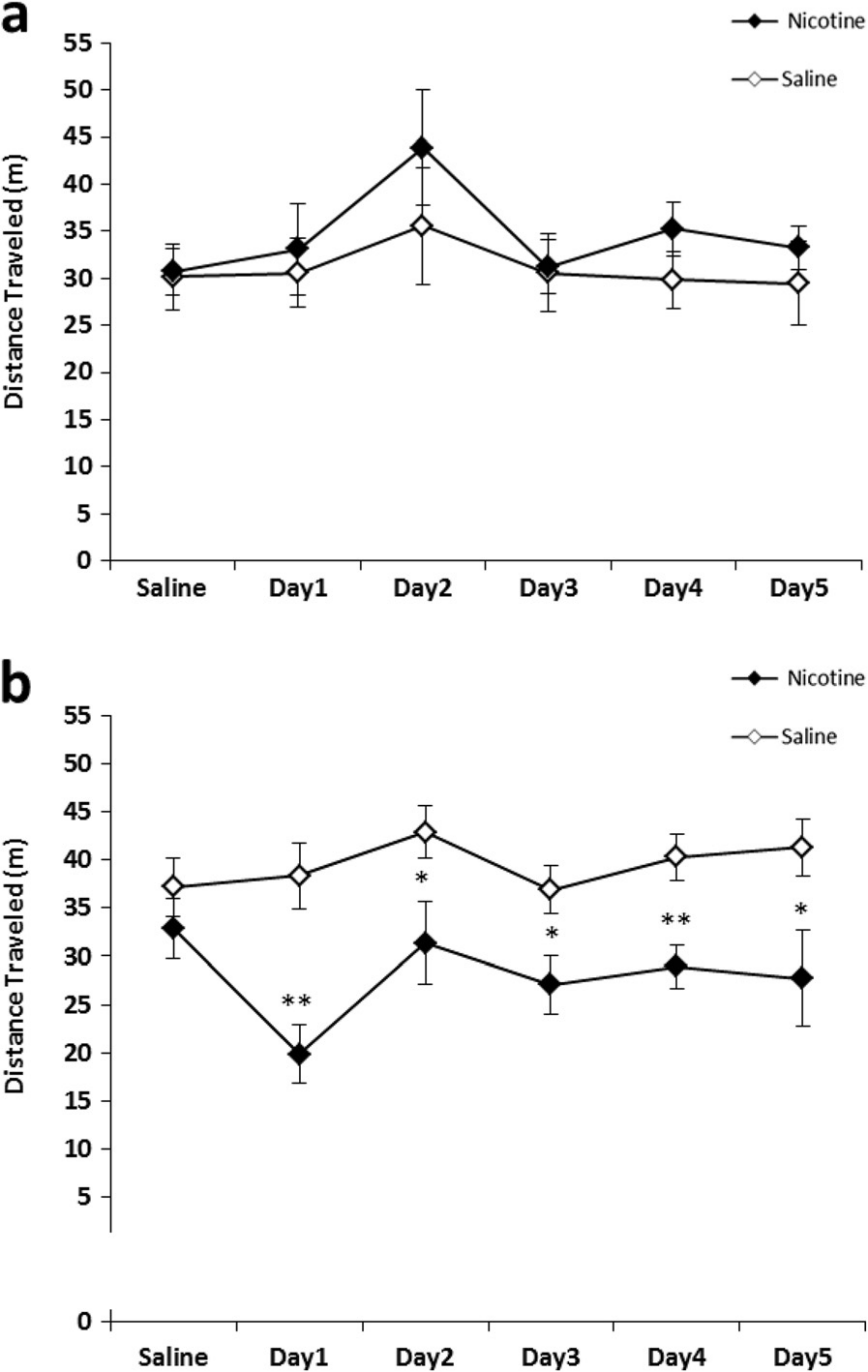
**Young adult locomotion following nicotine administration.** Nicotine (0.5 mg/kg) was administered for 5 consecutive days to *a/a* mice **(a)**, and *A*^*vy*^*/a* mice **(b)**. Data are presented as mean distance traveled in meters ± SE; *n* = 5–8 per group. * *p* < .05, ** *p* < 0.01 significantly different from saline-matched controls.

Overall 4-way repeated measures ANOVA showed a significant effect of Age x Genotype x Treatment (F_1, 46_ = 6.21, *p* < 0.05) on locomotor activity. Nicotine administration had no noticeable effect on locomotion in adolescent mice (Figure 1). In young adult mice, *a/a* mouse locomotion was not influenced by nicotine administration (Figure 2a) while *A*^*vy*^*/a* mice displayed hypolocomotion after nicotine treatment throughout the course of testing period (Figure 2b). Nicotine-treated young adult *A*^*vy*^*/a* mice were significantly different than their saline-matched controls on Day 1 (t_10_ = -4.06, *p* < 0.01), Day 2 (t_10_ = -2.26, *p* < 0.05), Day 3 (t_10_ = -2.52, *p* < 0.05), Day 4 (t_10_ = -3.44, *p* < 0.01), and Day 5 (t_10_ = -2.35, *p* < 0.05).

**Figure 3:**
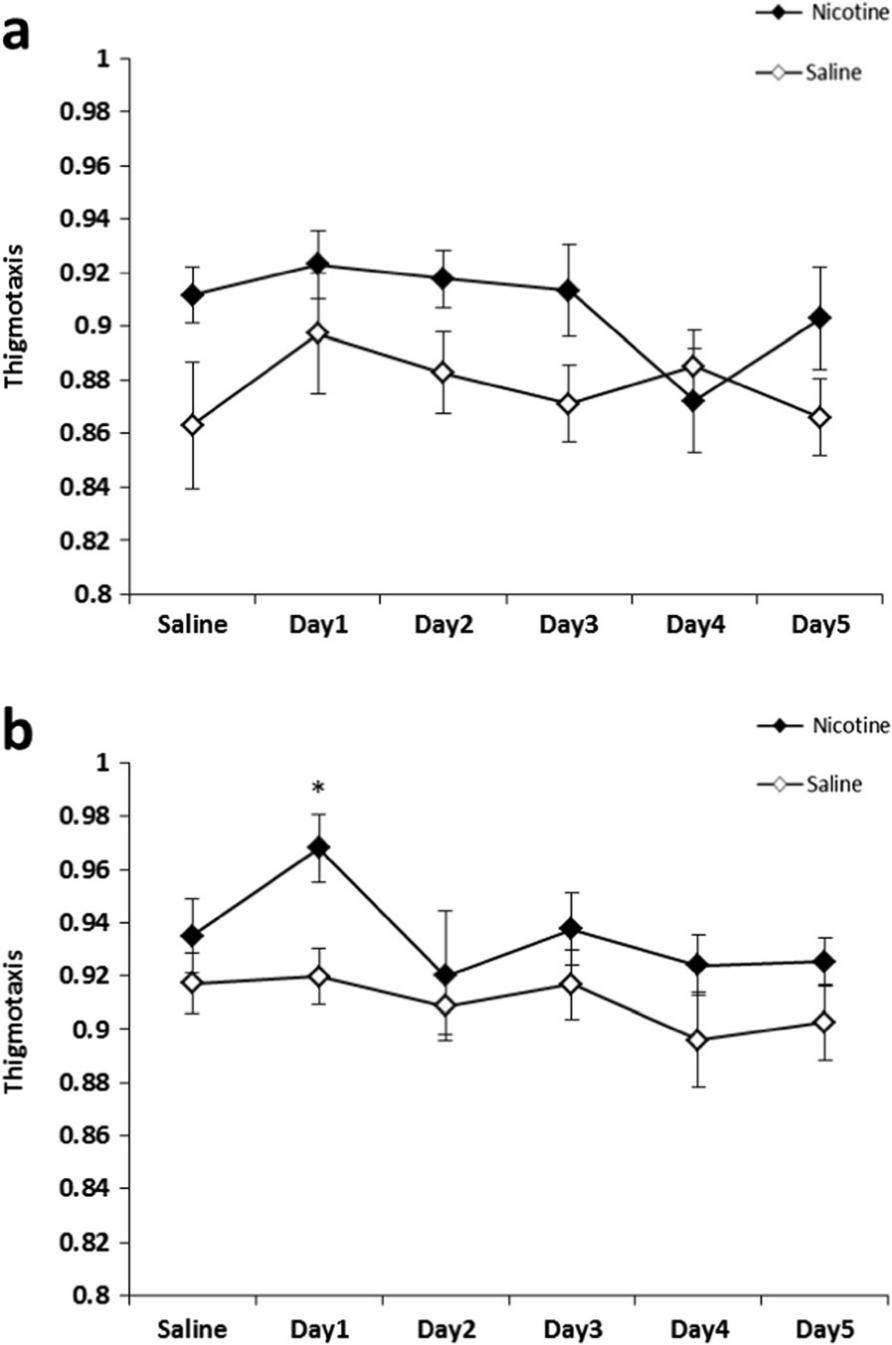
**Thigmotaxis in young adult mice following nicotine administration.** Nicotine (0.5 mg/kg) was administered for 5 consecutive days to *a/a* mice **(a)**, and *A*^*vy*^*/a* mice **(b)**. For graphical presentation, thigmotaxis was calculated as $1-\frac{\text{distance}\;\text{traveled}\;\text{outside}\;\text{center}\;\text{of}\;\text{apparatus}}{\text{total}\;\text{distance}\;\text{traveled}}$. Data are presented as mean ± SE; *n* = 5–8 per group. * *p* < 0.05 significantly different from saline-matched controls.

**Figure 4:**
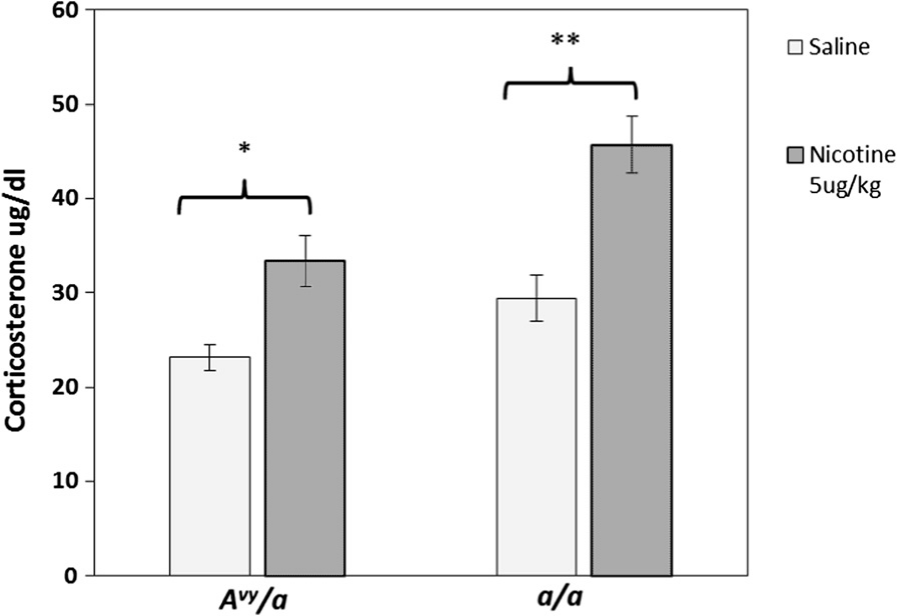
**Serum corticosterone following nicotine (0.5 mg/kg) administration in young adult *A***^***vy***^***/a *and *a/a *mice.***n* = 6–7 per group. *p < 0.05, ** *p* < 0.01 significantly different from saline-matched controls of the same genotype.

Nicotine did not induce thigmotaxis on any day in young adult *a/a* animals (Figure 3a). On most days, young adult *A*^*vy*^*/a* mice showed no difference from their *a/a* littermates in thigmotaxis (3-way repeated measures ANOVA *p* > 0.05). However, on Day 1, nicotine-treated young adult *A*^*vy*^*/a* mice displayed significant thigmotaxis (Figure 3b) as compared to saline-matched controls (t = 2.97, *p* < 0.05). There were significant main effects of Treatment (F_1, 21_ = 19.42, *p* < 0.001) and Genotype (F_1, 21_ = 9.58, *p* < 0.01) on serum corticosterone levels in young adult mice. Both *A*^*vy*^*/a* (t_9_ = -2.38, *p* < 0.05) and *a/a* (t_8_ = 3.84, *p* < 0.01) mice displayed increased corticosterone levels after nicotine administration in comparison to saline-matched controls (Figure 4). However, the magnitude of the corticosterone increase after nicotine administration was not significantly different between *A*^*vy*^*/a* and *a/a* mice.

### Discussion

Hypolocomotion after nicotine administration is common in mice at increasing doses, and is a potential indicator of sensitivity to the toxicity of nicotine [23,25]. That young adult *A*^*vy*^*/a* mice display such a robust locomotor depression that persists over the course of the testing period suggests that they are especially sensitive to nicotine administration at the 0.5 mg/kg dose. The observation that early adolescent *A*^*vy*^*/a* mice demonstrated no such effect may indicate that the emergence of the obesity phenotype in *A*^*vy*^*/a* mice, which occurs around the time of puberty [20], also corresponds with changes in sensitivity to the effects of nicotine administration. As it is thought that *A*^*vy*^*/a* obesity is driven to a large degree by Mc4r antagonism, it is plausible that Mc4r antagonism also plays a role in increased nicotine sensitivity.

However, adolescence in general represents a time of altered sensitivity to the effects of nicotine in mice. For example, adolescent male mice are more responsive than adults to the rewarding effects of nicotine as measured by conditioned place preference [26], and adolescent male mice are less sensitive than adults to the locomotor depression and hypothermia that occurs with higher doses of nicotine [27]. Thus, it may be that an altered response to nicotine in adolescent *A*^*vy*^*/a* mice obscures the influence the *A*^*vy*^ mutation and its downstream effects (e.g. Mc4r antagonism) have on nicotine sensitivity until adulthood.

It must also be recognized that the nature of the *agouti* mutation is complex, and differences in behavior between *A*^*vy*^*/a* and *a/a* animals do not necessarily involve ectopic expression of the *agouti* protein. For example, the *agouti (A)* gene is found on chromosome 2 [2], and a polymorphism in the coding portion of the α4 nicotinic receptor gene (also found on chromosome 2 in the mouse) has been observed to affect sensitivity to nicotine-induced seizures [28,29]. Nicotine-induced seizures are used as an indication of sensitivity to nicotine, and often strains that display greater susceptibility to seizures exhibit hypolocomotion at lower doses of nicotine than less-sensitive strains [30,31]. Thus, future work should explore the effects of the *agouti* mutation on other nearby genes, as such effects could potentially alter sensitivity to nicotine administration.

The thigmotaxis displayed by young adult male *A*^*vy*^*/a* mice after their first nicotine administration appears to indicate an anxiogenic response to initial nicotine administration at the 0.5 mg/kg dose. While the hypolocomotion and thigmotaxis of young adult *A*^*vy*^*/a* mice on the first day of treatment are likely related, it is important to note that the thigmotaxis does not appear to be a direct result of reduced locomotion (as this was controlled for in the calculation). Being placed in an open-field apparatus is considered a stressor for mice; it results in HPA axis activation and corticosterone secretion [22]. Nicotine administration in mice also results in corticosterone secretion [32]. Thus, we reasoned that the anxiety behavior in *A*^*vy*^*/a* mice might be associated with heightened levels of corticosterone secretion. A disparity in HPA axis activation, however, doesn’t appear to explain the differences in nicotine-induced thigmotaxis noted between young adult *A*^*vy*^*/a* and *a/a* mice. While *A*^*vy*^*/a* mice did display an elevation in serum corticosterone levels, the magnitude of the increase was similar to that displayed by *a/a* mice.

It is also possible that young adult *A*^*vy*^*/a* mice exhibit an enhanced sensitivity of HPA pathways and/or downstream targets in comparison to their *a/a* littermates, potentially as a result of Mc4r antagonism from *agouti* protein expression. A similar hypothesis has been suggested to explain a heightened sensitivity to the administration of corticotropin-releasing factor in Mc4r-null mice [9]. Lethal yellow (*A*^*y*^) mice, which also display excessive *agouti* expression and Mc4r inverse agonism, show greater reactivity to restraint and injection stress than B6, despite having comparable corticosterone levels after experiencing the stressor [33]. Thus, a compensatory enhancement of corticosterone sensitivity due to Mc4r antagonism seems plausible, but remains speculative until it can be tested with further research.

### Conclusion

Understanding the nature of aversive reactions to nicotine is important because the degree of aversion human smokers experience during their initial experience with smoking is a predictor of whether or not they continue to smoke [34,35]. The experiments presented here indicate that *A*^*vy*^*/a* mice may provide a unique model in which to study the aversive effects of nicotine and the role of the melanocortin system in the effects of nicotine. Additionally, the age-specific aspects of the results discussed above are of interest, as the vast majority of adult smokers begin smoking during adolescence [36]. Because adolescent *A*^*vy*^*/a* mice appear to be less sensitive to nicotine-induced hypolocomotion than adult *A*^*vy*^*/a* mice, the strain may represent a useful tool for studying differences between adult and adolescent responses to nicotine. Future research should attempt to replicate these findings; if successful it would be informative to further explore the effect using methods of nicotine administration that are more relatable to human nicotine consumption, such as intravenous self-administration. A more thorough exploration of the dosages that produce this aversive reaction would also be essential. Additionally, the interpretation of the anxiogenic effects seen here would benefit from additional experiments using alternative methodologies, such as the social interaction test or novel object exploration, in order to affirm the anxiogenic nature of the response. Finally, it may be valuable to explore the neurobiological basis of the strong reaction to nicotine administration seen here in young adult *A*^*vy*^*/a* mice.

## Competing interests

The authors declare that they have no competing interests.

## Authors’ contributions

MD, DV, and JG contributed to the conception and design of the study and the analysis and interpretation of the data. MD drafted the manuscript. All authors were involved in the collection and interpretation of the data and contributed to revisions to the final manuscript. All authors read and approved the final manuscript.
